# Qualitative analysis of cognitive and social congruence in peer-assisted learning from the medical students perspective

**DOI:** 10.1080/10872981.2020.1823619

**Published:** 2020-09-17

**Authors:** Michelle Kunc, Olivia Groom, Sally Barker, Nitish Nachiappan

**Affiliations:** School of Medicine, Imperial College London, London, UK

Dear Editor,

We were pleased to read the article by Loda et al. [1]. regarding the social and cognitive factors affecting peer-assisted learning in medical schools. As final-year medical students at Imperial College London, we have numerous personal experiences of both sides of the peer student-teacher dynamic spanning our time at medical school. We would like to share our experiences of student-led teaching from Imperial College’s Medical Education Society (MedED) and how social and cognitive factors come into play. Imperial MedED is run exclusively by students, organising lecture series, peer mentoring groups and mock examinations for clinical and preclinical medical students.

Teaching is invaluable in improving the students’ fluency and clarity of explanation, fostering the essential communication skills needed as a doctor. These skills come into play not only when teaching peers but are fundamental in the patient-doctor interaction [2,3]. The UK General Medical Council echo the importance of teaching competency for future doctors[4].

However, in our experience, many of our peers refrain from being student teachers due to the lack of confidence in their knowledge, as writing up and teaching a lesson plan can be intimidating. On the other hand, teaching can inspire the student to not only develop new skills, but also to consolidate previous learning and improve confidence in the topics being taught[2]. To promote the benefits of teaching, MedED runs a year-long teaching course in which students in the older years impart their knowledge and teaching techniques to preclinical students. This not only improves the students’ confidence but has the added benefit to ensuring consistency of teaching quality, as the younger students often go on to take up MedED teaching roles themselves as they progress through medical school.

Teaching also provides a controlled way for student teachers to receive feedback and improve on their delivery, helping reinforce the positive qualities and correct the poor aspects, further developing their teaching skills[5]. Students may feel more comfortable providing feedback for other students as they are more invested in the teaching and appreciate the effort put in by the student-teacher.

Furthermore, the student has access to pastoral support from the student teacher, gaining access to the ‘hidden curriculum’[3] allowing the student to ask questions and access notes in a more relaxed manner. This is particularly pertinent when peer teaching is done through clubs and societies, with many students we know citing this as one of the best environments to learn in. This is because you are learning from people you have an established personal relationship with and therefore, both sides are likely to be more invested in the teaching. We have also experienced that peer-assisted teaching is largely optional, meaning that students attend due to their own motivation, often in their personal time. As a result of this, often there is better engagement allowing the students to take ownership of their learning.

Overall, the social and cognitive factors outlined in the article by Loda et al. [1] which surround teaching are present widely throughout medical school. In our experience, these factors are pertinent in student-led teaching through university societies, which improve the quality of teaching and knowledge gained by students and student-teachers.
